# Association Between Lower-Limb Muscle Quality, Cognitive Function and Sarcopenia in Older Adults: Cross-Sectional Study

**DOI:** 10.3390/bioengineering13030265

**Published:** 2026-02-25

**Authors:** Arthur N. Arrieiro, Redha Taiar, Luana A. Soares, Jousielle M. Santos, Fabiana S. M. Pereira, Ângela A. Viegas, Leonardo A. C. Teixeira, Murilo X. Oliveira, Sueli F. Fonseca, Giovana A. Cordeiro, Adriana N. Parentoni, Vanessa A. Mendonça, Ana Cristina R. Lacerda

**Affiliations:** 1 Departamento de Fisioterapia, Universidade Federal dos Vales do Jequitinhonha e Mucuri, Diamantina 39100-000, MG, Brazilsoares.luana@ufvjm.edu.br (L.A.S.); leonardo.augusto@ufvjm.edu.br (L.A.C.T.);; 2 Programa de Pós-Graduação em Ciências da Saúde—PPGCS, Universidade Federal dos Vales do Jequitinhonha e Mucuri—UFVJM, Diamantina 39100-000, MG, Brazil; 3 MATériaux et Ingénierie Mécanique—MATIM, Université de Remis Champagne France, 51100 Reims, France; 4 Programa de Pós-Graduação em Reabilitação e Desempenho Funcional—PPGReab, Universidade Federal dos Vales do Jequitinhonha e Mucuri—UFVJM, Diamantina 39100-000, MG, Brazil

**Keywords:** aged, muscle strength, executive function, sarcopenia, walking speed

## Abstract

Background/Objectives: This cross-sectional study evaluated associations between physical function, cognitive function, sarcopenia, and the lower-limb Muscle Quality Index (MQI) in older adults. Methods: Patients with various MQIs (strength-to-mass ratios) were stratified into two groups. Physical-muscular aspects were assessed using body mass index (BMI), a Simple Questionnaire to Rapidly Diagnose Sarcopenia (SARC-F), and handgrip strength. Cognitive aspects were assessed using Mini-Mental State Examination (MMSE), Trail Making Test, Stroop Test, verbal fluency, and dual-task performance. Seventy-nine older adults (73 ± 9 years) of both sexes completed the tests. Results: BMI, SARC-F, handgrip strength, MMSE, Stroop Test (L1), semantic verbal fluency (animals), and dual-task outcomes differed between MQI groups (*p* ≤ 0.05) and were associated with MQI in regression analyses. In the adjusted model, BMI, handgrip strength, and dual-task walking time were independently associated with MQI (adjusted R^2^ = 0.514). Conclusion: Lower-limb MQI was associated with markers of sarcopenia and cognitive/dual-task performance in community-dwelling older adults.

## 1. Introduction

Aging is naturally associated with cognitive decline, a gradual process in which mental functions may decrease [1]. Age-related cognitive decline and early signs of dementia can be tracked through global assessments, such as the Mini-Mental State Examination (MMSE), which assesses global cognitive function, and specific tests that assess cognitive performance across multiple domains [2].

Sarcopenia is characterized by the progressive loss of muscle mass and strength associated with aging. This age-related decline may also be associated with decreased cognitive performance. However, evidence remains inconsistent, as there is no consensus on the definition and diagnostic criteria for sarcopenia [3]. Recently, the Global Leadership Initiative in Sarcopenia (GLIS) established a global conceptual definition of sarcopenia [4]. This definition highlights the assessment of specific muscle strength—a component of muscle quality—as a promising screening approach, determined by the ratio between strength and muscle mass [5].

Muscle quality refers to muscle tissue’s functional and structural characteristics and its capacity to generate force relative to muscle mass. It is defined by the ratio between muscle strength and muscle mass [6]. During aging, muscle mass and strength decrease at different rates, and this ratio can help characterize sarcopenia in this population [7]. The Muscle Quality Index (MQI) is a measure used to assess muscle tissue’s functional and structural efficiency, evaluating force output in relation to the quantity or size of muscle mass. There are several ways to quantify MQI, but there is yet to be a consensus, with the ratio between handgrip strength (HGS) and lean arm mass being the most widely used formula. However, the literature suggests that upper-body muscle quality does not differ substantially with age. In contrast, lower-body muscle quality deteriorates more rapidly with age and may be more indicative of physical disability [8].

The Muscle Quality Index (MQI) of the lower limbs (LLs), which is less commonly used than that of the upper limbs, is calculated as the ratio between lower-limb strength and leg lean mass [9]. Previous studies have used the ratio between the peak isokinetic torque of the quadriceps, obtained by isokinetic dynamometry, and the cross-sectional area of the thigh muscle, assessed by computed tomography. However, the utility of this approach is limited due to the high cost and complexity of the equipment [10]. The chair sit-to-stand test (SST) is a simple assessment used to evaluate strength, balance, and flexibility, and is frequently employed as a quick and easy measure of lower-limb muscle strength [11]. Dual-Energy X-ray Absorptiometry (DXA) is an objective and easy-to-interpret technology used to assess the overall and specific lean mass of body segments, such as the LLs [12]. Thus, the ratio between SST (lower-limb muscle strength) and DXA-derived performance (lower-limb lean mass) becomes a viable and applicable alternative for assessing lower-limb muscle quality in clinical and research settings.

Few studies have evaluated the possible relationship between muscle quality (MQ) and cognitive function, or its relevance as an aspect of sarcopenia in older adults, especially after the recent global definition of sarcopenia, which further emphasizes the MQI. In addition, most studies have focused on upper-limb MQ, and those that assessed lower-limb MQ used tools and devices that were difficult to access and results that were difficult to interpret [13,14]. It is, therefore, justified to carry out a study that aims to explore the relationship between MQI and cognitive aspects in older adults, considering that lower-limb function is an important component of mobility and independence, contributing to the maintenance of a healthy life. Accordingly, the objective of the present study was to evaluate the associations between lower-limb muscle quality, cognitive function, and sarcopenia in community-dwelling older adults.

## 2. Materials and Methods

This cross-sectional study was carried out in Diamantina, Brazil, with community-dwelling older adults from 2021 to 2024. It followed the guidelines of the Strengthening Reporting of Observational Studies in Epidemiology (STROBE) [15] tool for observational studies.

The sample consisted of community-dwelling older adults of both sexes residing in Diamantina. Participants were recruited through verbal invitations, leaflets, and other communication channels. Volunteers met the following criteria: (i) age ≥ 60 years; (ii) living in the community; (iii) able to read and write; and (iv) reporting current medication use.

The exclusion criteria were chronic cardiac (uncontrolled hypertension, cardiac arrhythmia, and pacemaker use), metabolic (uncontrolled diabetes mellitus and thyroid diseases), or inflammatory diseases (rheumatoid arthritis and severe hip or knee osteoarthritis) that made it impossible to participate independently in the proposed activities, and refusal to sign the Informed Consent Form (ICF). Educational level was recorded but not used as a covariate in this study; instead, it was used to confirm that participants could understand and complete the cognitive assessment tasks.

The study by Sui et al. (2022) [16] was used to estimate the sample size, considering an R^2^ of 0.24 (global cognitive function vs. muscle quality). Based on an effect size of 0.24, an alpha error of 0.05, and a power of 95% (0.95), and allowing for a 15% attrition rate, a total of 79 participants was required.

The present study was conducted at the Federal University of the Jequitinhonha and Mucuri Valleys (UFVJM) (Diamantina, Minas Gerais, Brazil), and included assessments of clinical, physical, functional, and cognitive outcomes.

All researchers (ANA, ACNP, LAS, VMTLA, and BVRO) were previously trained, and intra- and inter-examiner reliability for all tests and measurements was greater than 0.80.

The MQI was determined as the ratio between lower-limb muscle strength (the ability to generate force against resistance) and lower-limb muscle mass (muscle tissue volume). Lower-limb muscle strength was assessed using the sit-to-stand test (STS), and lower-limb muscle mass was assessed using DXA.

Participants were instructed to perform five sit-to-stand repetitions as quickly as possible without using their arms for support. The score was the time (s) required for participants to complete five repetitions [17]. Thus, shorter completion times indicate greater lower-limb strength and, consequently, higher muscle quality.

DXA was used to estimate body composition and nutritional status. This method has good precision, accuracy, and reproducibility. It is based on the measurement of three components (bone mineral content, fat mass, and lean mass), generating segment-specific data, such as lower-limb lean mass (g), which were recorded and used in the MQI formula [18].

Subsequently, the MQI was stratified into two groups to assess associations with physical and cognitive factors. Physical aspects were assessed using body composition, anthropometry, and muscle strength: body mass index (BMI), the Simple Questionnaire to Rapidly Diagnose Sarcopenia (SARC-F), and handgrip strength (HGS). Cognitive aspects were evaluated using the Mini-Mental State Examination (MMSE), the Trail Making Test (TMT), the Stroop Test, verbal fluency (VF), and dual-task (DT) assessment.

BMI was determined by dividing the individual’s mass (kg) by the square of their height (m) [19]. The SARC-F, a low-cost and moderately sensitive sarcopenia screening tool for predicting low muscle mass, was administered, with scores ranging from 0 to 10 [6]. HGS is widely used to indicate overall muscle strength and was measured using the Jamar^®^ dynamometer (Asimow Engineering Co., Los Angeles, CA, USA), following the protocol recommended by the American Society of Hand Therapists. For this assessment, participants were seated with the shoulder adducted and in neutral rotation, the elbow flexed at 90°, and the forearm and wrist in a neutral position, with slight wrist extension allowed (up to 30°). Three trials were performed, and HGS was calculated as the mean of the three measurements. A 1 min rest interval was provided between trials [20].

The MMSE is a specific assessment for detecting cognitive impairment in older adults, with a maximum score of 30 points and cutoff scores that depend on educational level [2]. The TMT is divided into two parts (A and B) and assesses visual attention and task switching [21]. The TMT-A consists of 25 consecutive numbers, and participants were instructed to connect the numbers in numerical order. The TMT-B consists of 12 successive numbers and 12 letters. The time to complete each test was recorded. The Stroop Test assesses inhibitory control under conditions of cognitive conflict [22]. The objective is to name the ink color in which the words are printed, ignoring the words’ meaning. Trials may be incongruent (the ink color differs from the word meaning) or congruent (the ink color matches the word meaning). Inhibitory control is quantified by the difference in responses between incongruent and congruent trials (the Stroop effect). VF requires participants to generate as many words as possible within a fixed period. It was divided into phonological fluency (words beginning with a specific letter: F, A, or S) and semantic/category fluency (words belonging to a semantic category, such as animals) [23].

The DT test was used to assess mobility and was adapted from Liao et al. (2019) [24]. Participants were asked to walk 10 m under three conditions: (1) walking at their preferred speed; (2) walking while performing serial subtraction starting from a random three-digit number (cognitive dual task); and (3) walking while carrying a tray with a glass of water (motor dual task). The primary task was walking, and the secondary tasks were serial subtraction or carrying a tray. Participants completed three trials under each condition, with 1 min intervals between trials, and were instructed to prioritize walking. The times required to complete each trial were recorded, and the outcome was calculated as the mean of the three trials [24].

Statistical analysis was performed using the Statistical Package for the Social Sciences version 22.0 (IBM, Armonk, NY, USA). Descriptive statistics were used to characterize the sample. Continuous variables were summarized as the mean ± standard deviation. Student’s *t*-test or the Mann–Whitney U test was used to compare groups, as appropriate. Univariate and stepwise multivariate linear regression analyses were performed to examine associations between the independent variables and the dependent (response) variable. The multivariate statistical model, even with its limitations due to model instability, was used to examine the joint effects of multiple variables and to control for potential confounders, ensuring that the effects collected are valid due to the variables of interest and not because of other underlying factors. In the multivariable model adjusted for age and sex, variables associated with MQI in the univariate analyses (*p* < 0.20) were included. Statistical significance was set at *p* < 0.05.

## 3. Results

At the end of the study procedures, 79 community-dwelling older adults (73 ± 9 years) of both sexes completed all assessments. Results are presented by comparing variables stratified by MQI (best vs. worst muscle quality) (Table 1).

BMI (*p* = 0.000), SARC-F (*p* = 0.005), HGS (*p* < 0.001), MMSE (*p* = 0.024), Stroop Test L1 (*p* = 0.037), VF-Animal (*p* = 0.032), DT-Walk (*p* = 0.004), DT-Cognitive (*p* = 0.030), and DT-Motor (*p* = 0.006) showed significant differences between MQI strata.

Furthermore, associations between variables were assessed using simple and multivariate linear regression models, with MQI as the dependent variable (Table 2).

Simple and multivariate linear regression models were used for *p*-values *p* < 0.20 to identify possible associations between the dependent variable (lower-limb MQI) and independent variables. BMI (R^2^ = 0.066; β = −0.258; *p* = 0.022), SARC-F (R^2^ = 0.093; β = 0.306; *p* = 0.006), HGS (R^2^ = 0.185; β = −0.306; *p* < 0.001), MMSE (R^2^ = 0.050; β = −0.225; *p* = 0.047), Stroop Test L1 (R^2^ = 0.057; β = 0.238; *p* = 0.035), VF-Animal (R^2^ = 0.066; β = −0.257; *p* = 0.022), DT-Walk (R^2^ = 0.349; β = 0.591; *p* < 0.001), DT-Cognitive (R^2^ = 0.238; β = 0.488; *p* < 0.001) and DT-Motor (R^2^ = 0.319; β = 0.565; *p* < 0.001) were associated with MQI.

In the multivariable model adjusted for age and sex, BMI (β = −0.233; *p* = 0.002), HGS (β = −0.267; *p* = 0.031), and DT-Walk (β = 0.457; *p* = 0.010) remained independently associated with MQI, and the model explained approximately 50% of the variance (adjusted R^2^ = 0.514).

## 4. Discussion

Lower-limb muscle quality was associated with overall body mass, MS, and sarcopenia, as well as global and specific aspects of cognitive function in community-dwelling older adults. In addition, higher lower-limb muscle quality was associated with better functional performance and cognition.

In older adults, there is strong evidence that MQI and sarcopenia are associated with cognitive performance. A cross-sectional study similar to the present study assessed the association between muscle quality and cognitive function [16]. The authors found an association between MQ and global cognition assessed by the MMSE, as well as with several cognitive domains. However, not all cognitive domains were associated with MQ after adjustment for age. These findings corroborate the results of the present study. Notably, MQ was determined using an upper-limb strength-to-mass ratio, and the study population consisted only of male participants. MQI can be defined in several ways; however, the most widely used approach is the ratio between upper-limb strength and mass [6]. Many studies use handgrip strength [13,14], which reflects overall muscle strength (MS) and is commonly used in sarcopenia screening [8]. Appendicular lean mass can be obtained through DXA, which estimates lean mass in the upper limbs [12]. However, upper-limb lean mass is less affected by advancing age, and the effects of sarcopenia are more pronounced in the lower-limb muscles [16]. A longitudinal study compared the validity of alternative measures of muscle quality in older men, using lower-limb muscle quality as a reference, and found similar results [25].

In relation to lower-limb MQ, this study found significant associations with sarcopenia and overall muscle strength, as assessed by SARC-F and HGS. The SARC-F questionnaire assesses functional limitations related to muscle strength and classifies individuals according to functional performance and risk of sarcopenia [26]. Handgrip strength is widely used to measure upper-limb strength, but it is also a robust proxy for overall muscle strength [13]. Thus, the findings of the present study are consistent with the purpose of these instruments in assessing strength and functional performance in older adults, as MQI stratification (better vs. worse muscle quality) corresponded to higher vs. lower muscle strength.

Cognitive measures (MMSE, Stroop Test, verbal fluency, and dual-task performance) showed significant differences across MQI strata. These findings are consistent with studies reporting an association between cognitive and functional performance, whereby older adults with better cognitive function tend to perform activities of daily living more easily, and those with functional limitations often show progressively poorer performance in cognitive tasks [27,28]. However, in the present study, MQ was not significantly associated with some cognitive domains. The Trail Making Test (TMT), for example, did not differ significantly between MQI groups. The Stroop Test showed a difference only for condition 1, which is the simplest. Language, assessed using the VF test, differed only for semantic fluency. A study evaluated the relationship among these three executive function tests and concluded that it is essential to adjust results for age, sex, and educational level, given their complexity and the potential influence of cognitive reserve [29].

An instrument that captures the association between functional and cognitive aspects is the DT test [24]. In the present study, individuals with better MQ completed the tests significantly faster than those with worse MQ, suggesting that MQ may play an important role in cognitive function during physical performance. However, because this was a cross-sectional study, further research is needed to confirm these findings. Measures of functional and cognitive performance showed significant differences across strata of the lower-limb MQI. Thus, assessing lower-limb muscle strength and quality may be important for evaluating functional performance and cognitive function [30]. Simple and practical tests of lower-limb strength and function, such as the chair SST [31], can be implemented in clinical settings to help screen for and potentially prevent cognitive decline in this population.

In addition, functional and cognitive performance were significantly associated with MQI. These associations were observed in the linear regression analyses and are consistent with prior studies showing that greater muscle mass and muscle quality are associated with a lower risk of sarcopenia, greater muscle strength, and better performance in reasoning, memory, and language, as well as shorter completion times on sustained and divided attention tasks [5,32,33,34].

Because muscle strength and muscle mass generally decline with advancing age, and differences are also observed between sexes, we additionally fitted a model adjusted for age and sex. As shown in Table 1, a higher proportion of men were in the better muscle quality group, whereas more women were in the poorer muscle quality group.

The age- and sex-adjusted model confirmed the associations of muscle strength and divided-attention task performance with lower-limb MQI and explained more than 50% of the variance (adjusted R^2^ = 0.514). These results strengthen the evidence that lower-limb muscle quality is associated with functional and cognitive performance in older adults; however, further studies—particularly longitudinal or interventional designs that account for age and sex—are needed to clarify these relationships.

Studies that assess functional and cognitive performance in older adults, aiming to identify and monitor early physical and brain changes, are valuable and should be increasingly encouraged. The identification and use of tests and instruments with practical clinical applicability, even when their predictive validity has not yet established, may help prevent progression from minor difficulties in daily tasks to physical and functional limitations, which can ultimately increase the risk of falls, disability, dependence for basic activities of daily living, and death. Although the present study aimed to explore the relationships between muscle quality and cognitive, functional, and sarcopenia-related outcomes, rather than to stratify these assessments, the lack of a direct evaluation of physical activity level and nutritional status, as well as the absence of sex-stratified analyses, represent limitations. Furthermore, given the cross-sectional design, caution is warranted when interpreting these findings, and causal inferences should not be made.

## 5. Conclusions

Using the lower-limb MQI as the dependent variable, our results align with the global conceptual definition of sarcopenia, suggesting that this approach is applicable for evaluating this condition and its associations with cognitive performance in older adults. These findings highlight the clinical and research relevance of MQI as a marker associated with sarcopenia and physical and cognitive performance, with potential utility for screening and monitoring. The results indicate that lower muscle mass, lower muscle strength, and the presence of sarcopenia, as well as poorer global, domain-specific, and dual-task cognitive performance, are associated with reduced lower-limb muscle quality in community-dwelling older adults in Brazil. Lower BMI, lower handgrip strength, and slower dual-task walking performance—after adjustment for age and sex—explained approximately 50% of the variance in lower-limb MQI in this sample.

## Figures and Tables

**Table 1:** Stratification of the sample in dulcis by lower-limb MQI.

Variable	Best Muscle Quality Lower SST/DEXA Ratio	Worst Muscle Quality Higher SST/DEXA Ratio	*p* Value
*N* (%)	40 (50.6)	39 (49.4)	_
Age (years)	75 ± 8.93	77 ± 8.78	0.113
Male, *n* (%)	13 (86.67)	2 (13.33)	_
Female, *n* (%)	29 (45.31)	35 (54.69)	_
Total Sex, *n* (%)	42 (53.16)	37 (46.84)	0.020 *
**Physical Aspects**			
BMI (kg/m^2^)	30.20 ± 4.48	25.97 ± 4.66	<0.001 *
SARC-F (pts)	1.35 ± 2.34	3.62 ± 4.36	0.005 *
HGS (Kgf)	33.31 ± 7.88	27.42 ± 4.42	<0.001 *
**Cognitive Aspects**			
MMSE (pts)	26.53 ± 2.57	24.62 ± 4.55	0.024 *
TMT-A (s)	75.45 ± 42.32	95.30 ± 76.23	0.155
TMT-B (s)	197.14 ± 92.85	198.79 ± 90.33	0.937
Stroop Test L1 (s)	46.38 ± 6.83	53.24 ± 19.18	0.037 *
Stroop Test L2 (s)	46.85 ± 8.92	49.55 ± 14.29	0.317
Stroop Test L3 (s)	43.63 ± 29.35	43.90 ± 35.65	0.970
Stroop Effect (pts)	0.97 ± 0.71	0.83 ± 0.69	0.406
VF-FAS (n)	30.56 ± 16.66	34.23 ± 11.91	0.264
VF-Animal (n)	16.45 ± 7.59	13.18 ± 5.54	0.032 *
DT-Walk (s)	10.89 ± 1.97	12.35 ± 2.40	0.004 *
DT-Cognitive (s)	13.63 ± 3.02	15.28 ± 3.61	0.030 *
DT-Motor (s)	12.33 ± 2.27	13.97 ± 2.87	0.006 *

MQI, Muscle Quality Index; LL, lower limb; BMI, body mass index; SARC-F, Simple Questionnaire to Rapidly Diagnose Sarcopenia; HGS, handgrip strength; MMSE, Mini-Mental State Examination; TMT, trail-making test; VF, verbal fluency; DT, dual task. Data are presented as mean (±SD) or n (%). * Significance level of 95% (*p* ≤ 0.05).

**Table 2:** Simple and multivariate linear regression.

Dependent Variable MQI of LL					
Independent Variable	β	*p* Valor	R^2^		
BMI	0.258	0.022 *	0.066		
SARC-F	−0.306	0.006 *	0.093		
HGS	0.431	<0.001 *	0.185		
MMSE	0.225	0.047 *	0.050		
Stroop Test L1	−0.238	0.035 *	0.057		
VF-Animal	0.257	0.022 *	0.066		
DT-Walk	−0.591	<0.001 *	0.349		
DT-Cognitive	−0.488	<0.001 *	0.238		
DT-Motor	−0.565	<0.001 *	0.319		
**Adjusted Model**			**β**	***p*** **-** **value**	**R^2^ adjusted**
					0.514
BMI			0.233	0.002 *	
HGS			0.267	0.031 *	
DT-Walk			−0.457	0.010 *	

MQI, Muscle Quality Index; LL, lower limb; BMI, body mass index; SARC-F, Simple Questionnaire to Rapidly Diagnose Sarcopenia; HGS, handgrip strength; MMSE, Mini-Mental State Examination; VF, verbal fluency; DT, dual task. * Significance level of 95% (*p* ≤ 0.05).

## Data Availability

The data supporting the findings of this study are available within the article. Additional information may be requested from the corresponding author.
